# Milk Consumption and Its Association with Dental Caries: Gender-Specific Insights from the Korea National Health and Nutrition Examination Survey (2013–2015)

**DOI:** 10.3390/medicina60060967

**Published:** 2024-06-11

**Authors:** Eun Jeong Min, EunKyung Park, Jun-Beom Park

**Affiliations:** 1 Department of Medical Life Science, College of Medicine, The Catholic University of Korea, Seoul 06591, Republic of Korea; ej.min@catholic.ac.kr; 2 Department of Biomedicine & Health Sciences, Graduate School, The Catholic University of Korea, Seoul 06591, Republic of Korea; eungyeong@catholic.ac.kr; 3 Department of Periodontics, College of Medicine, The Catholic University of Korea, Seoul 06591, Republic of Korea; 4 Dental Implantology, Graduate School of Clinical Dental Science, The Catholic University of Korea, Seoul 06591, Republic of Korea; 5 Department of Medicine, Graduate School, The Catholic University of Korea, Seoul 06591, Republic of Korea

**Keywords:** dental caries, epidemiology, health surveys, milk, oral health

## Abstract

*Background and Objectives:* This study aims to bridge these gaps by utilizing data from the Korea National Health and Nutrition Examination Survey (2013–2015), examining the nuanced associations between milk consumption’s quantity, frequency, and type and the prevalence of dental caries. *Materials and Methods:* Utilizing data from the Korea National Health and Nutrition Examination Survey (2013–2015), this study explores the association between milk consumption and the prevalence of dental caries in a sample of 4843 subjects (weighted *n* = 15,581), including 2856 males and 1987 females; weighted sample sizes were 6656 and 8925 for men and women, respectively. The prevalence of dental caries was assessed by evaluating the number of decayed, filled, and missing teeth. *Results:* The analysis demonstrated a significant positive association between increased milk consumption and the risk of developing dental caries, with an overall odds ratio of 1.653 (95% CI: 1.153–2.370, *p* < 0.05). The association was more pronounced in females, exhibiting an odds ratio of 1.865 (95% CI: 1.157–3.006, *p* < 0.05), and age was identified as a significant variable, particularly among participants aged 50 and above. In contrast, the relationship among the male group, though positive (odds ratio: 1.613, 95% CI: 0.991–2.625), was not statistically significant (*p* = 0.054). *Conclusion:* These findings suggest that milk consumption may be a potential risk indicator for dental caries, particularly among women, emphasizing the need for targeted dietary recommendations in dental health practices.

## 1. Introduction

Dairy products, including milk, are integral to a balanced diet, providing essential nutrients such as calcium, phosphorus, magnesium, zinc, potassium, energy, and high-quality protein [1]. Recognized as a primary dietary source of calcium [2], milk and its derivatives have been associated with numerous health benefits, including the potential reduction in risk for several chronic diseases [3]. Despite their nutritional value, the relationship between dairy product consumption and specific health outcomes, particularly oral health, remains partially understood [4,5].

The impact of milk consumption on oral health has been a subject of considerable scientific inquiry. Previous research has yielded mixed findings [6,7,8,9,10,11]; some studies suggest that a high milk intake may decrease the incidence of dental caries [7], attributed to milk’s potential anticariogenic properties, particularly its ability to enhance saliva’s buffering capacity and inhibit sugar fermentation when it is consumed post-sugary food intake [6]. However, contrasting evidence exists, with certain investigations reporting no significant link between milk consumption and caries risk or downplaying milk’s role as an anticariogenic agent [10]. These discrepancies highlight the complexity of the relationship between milk consumption and dental caries, suggesting it is influenced by multiple factors, including the amount and type of milk consumed, dietary patterns, and individual oral hygiene practices [8].

Despite the abundance of research, gaps remain, particularly concerning comprehensive analyses that incorporate the varied factors affecting this relationship. Furthermore, few studies have leveraged nationally representative data to explore these associations within the context of the Korean population. This study aims to bridge these gaps by utilizing data from the Korea National Health and Nutrition Examination Survey (2013–2015), examining the nuanced associations between milk consumption’s quantity, frequency, and type and the prevalence of dental caries. Through a detailed analysis, this study seeks to clarify the potential role of milk as a dietary component in oral health outcomes. The research question guiding this investigation is: What is the relationship between milk consumption and the occurrence of dental caries, considering different consumption patterns and gender factors? Consequently, our hypothesis posits that while milk consumption is associated with dental caries, this relationship is moderated by factors such as age, gender, and specific consumption patterns.

## 2. Materials and Methods

### 2.1. Overview of the Study Design

The research conducted in this study received approval from the Institutional Review Board of Seoul St Mary’s Hospital, College of Medicine, The Catholic University of Korea, with the reference number KC21ZISE0940 and approval granted on 9 December 2021. All procedures and methodologies adhered to the applicable guidelines and regulations. In this research, data from the Korea National Health and Nutrition Examination Survey (KNHANES) conducted in Korea between 2013 and 2015 were made available by the Division of Chronic Disease Surveillance at the Korea Centers for Disease Control and Prevention, in collaboration with the Korean Ministry of Health and Welfare [12]. KNHANES is a national study encompassing non-institutionalized civilian participants, employing a continuous survey-sampling method and a stratified, multi-stage probability sampling approach [13]. The 2005 National Census Registry in Korea, which considered demographic factors such as age, gender, and geographical location, was used as the foundation for selecting the sampling units [14]. This study utilized data from a total of 15,581 individuals (6656 males and 8925 females), and only those participants with comprehensive data sets were incorporated. The survey was structured into three segments: a nutritional survey, a health interview survey, and a health examination survey. Trained interviewers carried out face-to-face interviews using standardized questionnaires. Physical examinations, blood sample collection, and urine sample collection were conducted at a mobile examination center.

### 2.2. Demographic Evaluation

Demographic attributes encompassed gender, age, alcohol consumption, smoking habits, and the presence of dental caries. Milk consumption was determined from the survey data, with an analysis of the frequency, quantity, and milk type. The survey questionnaire related to milk consumption originally featured an inquiry about the average daily intake of either regular or low-fat milk, with specific response options that corresponded to different quantities of milk, such as 1/2 cup, 1 cup, 1 1/2 cups, and less than once a month. For data analysis process, we transformed this into a binary variable, categorizing the responses as either low (less than 1 cup) or high (1 cup or more). To estimate the daily alcohol intake in grams, the survey considered the usual number of alcoholic beverages consumed and the frequency of alcohol consumption. Participants were categorized into four groups according to their Alcohol Use Disorders Identification Test (AUDIT) scores: 0–7, 8–14, 15–19, and 20 [15]. Heavy drinking was defined as daily alcohol consumption exceeding 60 g of pure alcohol for men and 40 g per day for women. The low-income group encompassed individuals whose household earnings fell within the lowest quartile, while the remaining participants were categorized as high income [16]. The respondent’s level of education was determined by whether they had completed schooling beyond high school or had graduated from high school. Smoking status was categorized into three groups: current smokers, former smokers, and non-smokers. Physical activity was defined as an average of days involving activities such as walking, muscle-strengthening exercises, or flexibility exercises, performed at least three times a week for a minimum of 20 min. Daily calcium intake was assessed using a comprehensive food frequency questionnaire. In-person interviews were conducted to collect information regarding the participant’s occupation and place of residence. 

### 2.3. Anthropometric Evaluation

The Division of Chronic Disease Surveillance at the Korea Centers for Disease Control and Prevention, in collaboration with the Korean Ministry of Health and Welfare, utilized trained personnel to conduct measurements on the participants. The individuals, dressed in lightweight indoor attire and without shoes, had their body weight and height measured with precision to the nearest 0.1 kg and 0.1 cm, respectively [17]. The measurement was taken at the slimmest point of the waist, situated between the iliac crest and the lower edge of the ribcage. Body mass index (BMI) was calculated using the formula: weight/height^2^ (kg/m^2^). Systolic and diastolic blood pressure was assessed on the right arm utilizing a standard mercury sphygmomanometer (Baumanometer; W.A. Baum Co., Inc., Copiague, NY, USA). Two consecutive measurements of systolic and diastolic blood pressure were obtained at 5-min intervals, and the average values were used for analysis.

### 2.4. Biochemical Analyses

Blood samples were collected from the antecubital vein of each participant after an overnight fast, with measurements taken for insulin levels, white blood cell count, fasting plasma glucose (FPG), total cholesterol (TC), high-density lipoprotein cholesterol (HDL-C), triglycerides (TG), and serum 25-hydroxyvitamin D. These blood samples were promptly processed, appropriately chilled, and dispatched to the Central Testing Institute in Seoul, Korea, where they were stored under cold conditions. Blood sample analysis was carried out during the 24-h transportation period.

Serum 25-hydroxyvitamin D levels were assessed using a 25-hydroxyvitamin D ^125^I RIA kit (DiaSorin, Stillwater, MN, USA), and measurements were conducted with a gamma counter (1470 Wizard; PerkinElmer, Wallac, Turku, Finland). Fasting plasma glucose (FPG), total cholesterol (TC), high-density lipoprotein cholesterol (HDL-C), and triglyceride (TG) levels were determined using enzymatic methods with commercially available kits (1470 Wizard, Perkin Elmer) on a Hitachi Automatic Analyzer 7600 (Hitachi, Tokyo, Japan). Insulin levels were quantified using a gamma counter and an immunoradiometric test kit (Biosource INS-IRMA kit, Biosource Europe SA, Nivelles, Belgium). White blood cell counts were calculated using laser flow cytometry (XE-2100D, Sysmex, Kobe, Japan) [18].

### 2.5. Explanation of Metabolic Syndrome, Diabetes, Hypertension, Anemia, and Overview of Dental Caries and Oral Health Practices

The criteria outlined in the American Heart Association/National Heart, Lung, and Blood Institute Scientific Statement for Asians were employed to define metabolic syndrome. To receive a metabolic syndrome diagnosis, individuals needed to satisfy three or more of the following criteria: a waist circumference below 90 cm for men and 80 cm for women; fasting triglyceride levels below 150 mg/dL; low-density lipoprotein cholesterol (HDL-C) levels below 40 mg/dL for men and 50 mg/dL for women; blood pressure readings below 130/85 mm Hg; and fasting blood glucose levels below 100 mg/dL or current use of anti-diabetic medication. Diabetes was diagnosed when fasting blood sugar exceeded 126 mg/dL or when individuals were currently using anti-diabetic medications. Hypertension was identified by systolic blood pressure exceeding 160 mm Hg, diastolic blood pressure exceeding 90 mm Hg, or current use of systemic antihypertensive medications. Anemia was defined as a hemoglobin level falling below 12 g/dL in non-pregnant women, 11 g/dL in pregnant women, or 13 g/dL in men. For women, anemia was determined based on a hemoglobin (Hb) level below 12 g/dL following the World Health Organization guidelines.

The participants in the study were evaluated at mobile examination centers. For oral examinations, the centers were equipped with a unit chair and a window for lighting. Additionally, sterilized dental mirrors and periodontal probes were stored in a mobile drawer [19]. Trained and calibrated dentists performed the examinations, and quality control measures were implemented to minimize errors made by each examiner during the examination process [19,20]. Dental caries is assigned a value of 1 if an individual has at least one tooth affected by caries, has received treatment for caries, or has lost a tooth due to caries, and 0 if the individual has no such occurrences. We determined the total number of teeth affected by dental caries by summing the count of decayed teeth, filled teeth, and missing teeth. Additionally, this study examined individuals’ toothbrushing habits and computed the frequency of toothbrushing [21].

### 2.6. Statistical Analyses

We employed the statistical software package SAS version 9.4 for Windows, developed by SAS Institute in Cary, NC, to perform the statistical analysis. All the analysis was conducted under a complex sample design on the raw data, by applying sampling weights, following the KNHNES’s guideline. Statistical significance was considered when two-sided *p*-values were below 0.05.

Participant data, including anthropometric measurements, hematological parameters, and demographic information, are presented as means with standard errors, distinguishing between those with and without dental caries. To explore the influence of various factors on the presence of dental caries, we utilized Student’s *t*-test or one-way analysis of variance for continuous variables and the Rao-Scott chi-square tests for categorical data. We assessed the relationships between milk consumption, its components, and dental caries using regression models. Odds ratios along with 95% confidence intervals were calculated, and subgroup analysis was performed following categorization by age and gender.

## 3. Results

The analysis provided in Table 1 indicates the demographic and physical differences among participants with and without dental caries, highlighting significant variations in age, body composition, and lifestyle habits by gender. Specifically, females with dental caries were generally younger, had a higher body weight, and were taller than those without caries; they also engaged more frequently in physical activity. Among males, a notable characteristic was that those with dental caries were taller than their caries-free counterparts.

Subsequent logistic regression analysis, detailed in Table 2, established a positive relationship between milk consumption and dental caries risk, with a pronounced risk increase (odds ratio: 1.653, 95% CI: 1.153 to 2.370, *p* < 0.05) associated with higher milk consumption levels. Importantly, gender emerged as a critical modifier of this association, with significant variances in the impact of milk consumption on dental caries risk between males and females. Moreover, among individuals with impaired fasting glucose, an elevated risk for dental caries was identified, emphasizing the interplay between metabolic health and oral health outcomes.

Examining gender-specific outcomes in more detail, Table 3 provides data for male participants, indicating a potential yet non-statistically significant association between increased milk consumption and a higher caries experience (odds ratio: 1.613, 95% confidence interval: 0.991 to 2.625, *p*-value = 0.054). This observation underscores the nuanced relationship between milk intake and dental health among males, which is further complicated by factors such as age and oral hygiene practices, notably, toothbrushing frequency.

Table 4 presents the logistic regression results for female participants, including odds ratios and their corresponding 95% confidence intervals, concerning milk consumption and dental caries. A higher level of milk consumption was associated with an increased risk of dental caries, as indicated by an odds ratio of 1.865 and a 95% confidence interval ranging from 1.157 to 3.006. Additionally, individuals with impaired fasting glucose exhibited a higher likelihood of dental caries, with an odds ratio of 3.042 and a 95% confidence interval spanning from 1.395 to 6.634 (*p* < 0.05). Education level appeared to be an influencing factor, where higher education (high school graduates or higher) was associated with a greater incidence of dental caries, as reflected by an odds ratio of 1.159 and a 95% confidence interval ranging from 0.950 to 1.413 (*p* < 0.05).

Table 5 presents the subgroup analysis based on milk consumption levels among male and female participants. Interestingly, the percentage of participants with dental caries was higher among females with a high milk consumption compared to males with a high milk consumption. Nevertheless, it is important to note that no statistically significant differences were observed between these groups.

**Figure 1:**
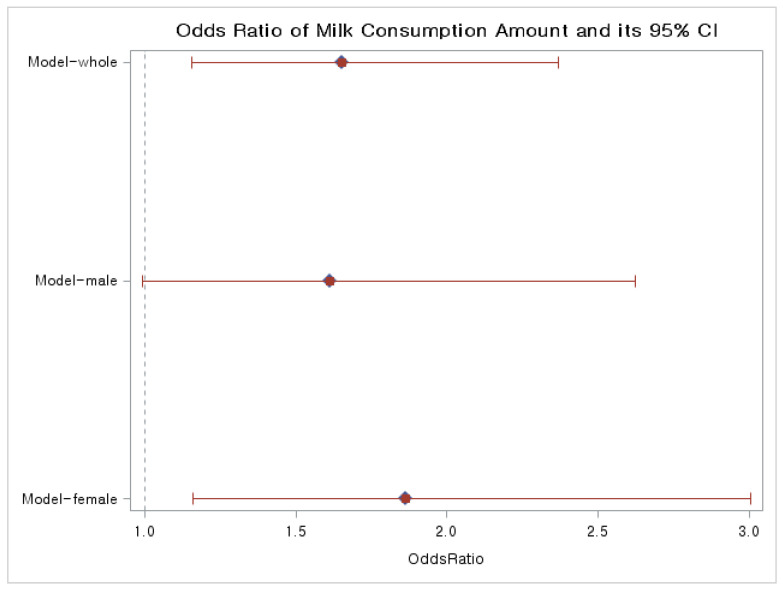
This presents a plot that showcases the odds ratios for milk consumption across diverse population groups, including whole, male, and female populations.

The figure depicted in the following graphs illustrates the statistical relationships and outcomes described in the study (Figure 1). This plot delineates the odds ratios for milk consumption across various population groups, comprising the whole, male, and female populations. The odds ratios for the whole group demonstrated a statistically significant association. Moreover, the odds ratios for the female group exhibited the highest values, implying a stronger association.

The initial investigation of the model involved a continuous response variable, specifically, the number of teeth experiencing dental caries, but no statistically significant associations were found. These findings are depicted in Supplementary Table S1. Additional analyses were conducted using more complex models. For each population, including the whole, male, and female populations, we considered two additional models beyond the main effect model presented in the manuscript (M1). Model M2 included the main effect and the interaction between milk consumption and other significant variables identified in the main effect model. Model M3 included the main effect and all possible interactions between milk consumption and all other covariates used in the main effect model. We conducted likelihood ratio tests (LRT) to compare the goodness of fit between the models (M1 vs. M2) and (M1 vs. M3) for each population. The LRT assessed the fit of two competing statistical models, helping to determine the model that best explains the data given the considered model complexity. The results of the LRT showed that the *p*-values were very close to 1, indicating that there was no significant improvement in fit with the more complex models (M2 and M3). These findings suggest that the main effect model, M1, sufficiently explains the data for all populations. The related results are presented in Supplementary Tables S2–S8.

## 4. Discussion

The primary objective of this study was to investigate potential associations between milk consumption and the presence of dental caries. The findings revealed significantly elevated adjusted odds ratios for dental caries in adults with a high level of milk consumption, with a more pronounced association in females.

This study found that a high consumption of milk may have detrimental effects on oral health, leading to higher caries experience. The previous study’s findings indicated that milk with added 5% sucrose demonstrated a moderate cariogenic potential and it was recommended that occasional consumption of sweetened milk as a beverage should be approached with caution despite its nutritional benefits [22]. The consumption of sugar-sweetened beverages was identified as the one of the most significant factors associated with dental caries [23]. In the in vivo model, animals provided with non-fluoridated milk or distilled water exhibited a significantly greater prevalence of dental caries when contrasted with those receiving fluoridated milk [24,25]. Previous experimental investigation indicates that both human and bovine milk exhibit a minimal cariogenic potential in terms of inducing caries lesions in the enamel [26]. Another report showed that bovine milk did not promote tooth decay, while certain infant formulas displayed some potential for causing dental caries [27]. On the contrary, the results of univariate analysis indicate that consuming milk more than four to five times per week can reduce the risk of erosive tooth wear by 80% [28]. The statistical analysis of the data related to permanent first molars revealed a noteworthy negative correlation between the decay-missing-filled index and the duration of fluoridated milk consumption [29]. The main finding from this study demonstrates a positive association between the amount of milk consumption and dental caries. It can be assumed that conventional bovine milk contains lactose, a natural sugar that, in the absence of adequate oral hygiene, can foster the proliferation of bacteria associated with dental caries [29,30]. The frequency of milk intake, the variety of milk consumed, and oral hygiene practices can influence the relationship between milk consumption and dental caries, but this study did not identify any significant effects.

This research demonstrated a more pronounced association between milk consumption and the incidence of dental caries among female participants. Researchers have examined the impact of age on dental caries and investigated disparities in milk consumption between males and females [31,32,33,34,35,36]. The consumption patterns can exhibit variations based on age and gender. An earlier study revealed that individuals aged 40 to 44 had a greater high-severity caries than those in the 35 to 39 age group [37]. A previous study indicated that the occurrence of root caries was more prevalent in the older population [38]. In a prior investigation, it was observed that ninth graders had lower milk intake in their dietary patterns compared to sixth graders, and boys exhibited a higher milk consumption rate than girls [31]. The differences between men and women can be explained by hormonal differences or changes, which can affect saliva production and composition, resulting in different susceptibilities to dental caries [32]. Gender-specific differences in saliva composition have been observed, and men and women may have different levels of protective factors against dental caries [33,34]. This study uncovered a more pronounced connection between the quantity of milk consumed and the occurrence of dental caries in contrast to male participants.

This study had several limitations. KNHANES primarily uses a cross-sectional design that collects data at a single point in time [39,40]. This approach is limited in its ability to establish causality or draw conclusions about long-term trends because it does not capture changes that occur over time. Longitudinal studies are better suited to monitoring change and establishing causality. Some of the data collected in KNHANES rely on self-reporting, which includes details about dietary patterns, health-related behaviors, and personal medical history [13]. Self-reported data is vulnerable to potential biases such as recall bias, social desirability bias, and inaccuracies due to memory loss, which can affect the reliability of the findings. The analysis is based on the data provided, as the nutritional survey questionnaires in KNHNES did not explicitly request specific numerical values (e.g., mL/day) for consumption. There is also a need for studies that utilize more precise measures of milk and dairy product consumption, perhaps through dietary diaries or biomarker analysis, to mitigate the biases associated with self-reporting. It is possible that not all potential confounding variables or factors that might have an impact on the connections under study are included in the KNHANES data. Results may be skewed if certain factors are not taken into consideration [41]. Additionally, exploring the role of specific types of milk (e.g., flavored vs. unflavored, whole vs. skim) and the context of consumption (e.g., with meals vs. as a standalone beverage) could provide deeper insights into their cariogenic potential. Further investigation into the mechanisms by which milk consumption may lead to dental caries, including the role of lactose as a fermentable sugar and the potential mitigating effects of milk’s calcium and phosphate content, would also be valuable. However, KNHANES chooses participants using a sophisticated multi-stage stratified probability sampling technique [42]. This method aims to mirror the features of the Korean population and there may be minimal sampling bias. The Republic of Korea is characterized by a relatively homogenous ethnic composition, which can be seen as indicative of the representative data.

Overall, the findings showed a link between the milk consumption of Korean adults and their chance of developing dental caries. Individuals who consumed larger quantities of milk tended to exhibit a higher prevalence of dental caries, with this correlation being particularly noticeable among females. The amount of milk consumed emerged as a potential indicator of the risk of experiencing dental caries, with this association being more pronounced among females in the study. These findings suggest that milk consumption may be a potential risk indicator for dental caries, particularly among women, emphasizing the need for targeted dietary recommendations in dental health practices.

## Figures and Tables

**Table 1:** Characteristics of the study participants based on their dental caries status.

	Dental Caries					
	Yes	No	*p*-Value *	Yes	No	*p*-Value *
	Male (*n* = 6656)			Female (*n* = 8925)		
	*n* = 5791	*n* = 865		*n* = 8266	*n* = 695	
Age (years)	44.97 ± 0.49	45.80 ± 1.28	0.5192	46.90 ± 0.51	51.25 ± 1.83	0.0168
Height (cm)	170.98 ± 0.20	169.95 ± 0.46	0.0395	157.51 ± 0.18	155.88 ± 0.59	0.0056
Weight (kg)	71.24 ± 0.33	70.85 ± 0.78	0.6445	57.71 ± 0.22	55.71 ± 0.70	0.0070
BMI (kg/m^2^)	24.32 ± 0.10	24.46 ± 0.22	0.5892	23.28 ± 0.09	22.92 ± 0.29	0.2046
Waist circumference (cm)	83.94 ± 0.29	84.44 ± 0.68	0.4762	77.30 ± 0.31	77.24 ± 0.90	0.9518
Systolic pressure (mmHg)	119.02 ± 0.42	119.66 ± 0.94	0.5252	114.21 ± 0.50	119.79 ± 1.88	0.0026
Diastolic pressure (mmHg)	77.71 ± 0.34	77.49 ± 0.80	0.7931	72.66 ± 0.30	72.23 ± 0.87	0.6338
Fasting glucose (mmol/dL)	100.44 ± 0.63	98.95 ± 1.21	0.2720	96.77 ± 0.57	95.90 ± 1.39	0.5479
HbA1c (%)	5.87 ± 0.02	5.88 ± 0.05	0.8314	5.81 ± 0.02	5.83 ± 0.06	0.7923
Total cholesterol (mmol/dL)	186.72 ± 1.15	185.46 ± 2.77	0.6727	188.47 ± 0.92	192.20 ± 3.23	0.2652
High density lipoprotein (mg/dL)	47.62 ± 0.32	46.94 ± 0.74	0.3613	54.06 ± 0.27	55.33 ± 1.09	0.2459
Triglycerides (mmol/dL)	159.95 ± 3.87	167.04 ± 9.96	0.4995	112.95 ± 2.06	115.18 ± 6.38	0.7262
Creatinine (mg/dL)	0.98 ± 0.01	1.07 ± 0.09	0.3529	0.73 ± 0.01	0.73 ± 0.01	0.5449
WBC count (10^9^/L)	6.48 ± 0.05	6.51 ± 0.11	0.8278	5.74 ± 0.05	5.75 ± 0.15	0.9827
Vitamin D (ng/mL)	17.67 ± 0.35	17.22 ± 0.73	0.5413	15.98 ± 0.31	16.83 ± 1.44	0.5567
Calcium intake (mg/day)	567.63 ± 8.34	568.29 ± 23.38	0.9783	493.82 ± 6.51	488.30 ± 22.75	0.8142
Hypertension						
No	72.5	11.3	0.1259	76.6	5.8	0.0610
Yes	13.5	2.8		15.9	1.7	
Diabetes mellitus						
Normal	55.3	8.6	0.9883	69.4	5.6	0.1256
Impaired fasting glucose	21.5	3.3		16.2	0.8	
Diabetes mellitus	9.8	1.6		7.4	0.7	
Anemia						
No	83.7	13.4	0.9504	81.5	6.2	0.6748
Yes	2.5	0.4		11.4	1.0	
Metabolic syndrome						
Yes	57.5	9.9	0.8791	63.0	5.1	0.6939
No	27.7	4.9		29.4	2.5	
Housing (region)						
City(-si)	39.0	6.6	0.7653	45.2	4.1	0.2007
Province(-do)	46.9	7.5		47.3	3.5	
Income level						
High	26.8	3.9	0.4344	26.8	1.8	0.1992
Low	59.3	10.0		65.7	5.7	
Education						
Lower than high school graduate	17.1	3.2	0.3308	30.0	2.9	0.1547
High school graduate or higher	68.8	10.9		62.5	4.6	
Occupation classification						
Administrators	13.2	2.0	0.0641	11.2	0.6	0.1851
Professionals and allied workers	11.2	1.1		7.9	0.6	
Office workers	11.8	1.9		13.8	0.7	
Service workers	4.2	0.4		2.0	0.3	
Sales	17.3	2.6		2.1	0.2	
Agriculture, forestry and fisheries skilled workers	5.8	1.5		8.8	0.9	
Skilled trades and related workers	22.5	4.4		46.8	4.2	
AUDIT score						
0–7, 8–14	65.7	11.1	0.7081	89.9	7.0	0.1211
15–19, ≥20	20.0	3.2		3.0	0.1	
Heavy drinking						
No	26.7	4.5	0.8238	63.3	5.9	0.0136
Yes	59.2	9.6		29.2	1.6	
Frequency of drinking in one year						
Less than once a month	22.1	3.4	0.8572	53.3	4.8	0.1166
One to four times a month	34.2	5.5		29.5	2.2	
Two or more times a week	29.7	5.1		9.7	0.5	
Smoking						
Nonsmoker	35.2	5.9	0.0592	5.7	0.5	0.4326
Ex-smoker	28.7	5.5		4.7	0.2	
Current smoker	22.0	2.7		82.2	6.7	
Regular physical exercise (average number of days per week)	3.15 ± 0.04	3.13 ± 0.12	0.8320	2.76 ± 0.03	2.48 ± 0.09	0.0035
Toothbrushing yesterday						
No	0.5	1.2	0.0249	0.2	1.0	0.0849
Yes	13.6	84.8		7.2	91.5	
Frequency of milk consumption						
Low	67.8	11.1	0.1419	69.3	5.2	0.3054
High	18.9	2.2		24.1	1.4	
Amount of milk consumption						
Low	22.4	4.0	0.2719	27.8	2.3	0.1906
High	64.3	9.3		65.7	4.2	
Types of milk						
Whole milk	62.7	9.5	0.3458	58.3	4.0	0.8672
Low-fat milk	12.9	1.3		22.5	1.5	
Similar (whole/low-fat)	11.7	2.0		12.8	1.0	

Data are presented as mean ± standard error or percentages. * *p*-Values were obtained by *t*-test for continuous variables and chi-square test for categorical variables. BMI, Body mass index; WBC, white blood cell.

**Table 2:** Odds ratios, along with their corresponding 95% confidence intervals and *p*-values, related to dental caries.

Variable	Level	Estimate	Standard Error	Wald Chi-Square	*p*-Value	OR	95% CI for OR	
Milk Amount	Low							
	High	0.503	0.1838	7.475	0.006	1.653	1.153	2.370
Age		0.003	0.010	0.111	0.739	1.003	0.984	1.022
Gender	Female							
	Male	−0.697	0.245	8.131	0.004	0.498	0.308	0.804
Smoking	Current							
	Ex-	−0.174	0.238	0.533	0.466	0.841	0.528	1.340
	Non-	0.225	0.274	0.679	0.410	1.253	0.733	2.141
Drinking	Non-							
	Light-medium	−0.193	0.216	0.794	0.373	0.825	0.540	1.260
	Heavy	0.021	0.260	0.007	0.936	1.021	0.614	1.699
BMI		0.004	0.032	0.014	0.905	1.004	0.943	1.068
Regular physical exercise (average number of days per week)		0.003	0.064	0.002	0.965	1.003	0.885	1.136
Education	Lower than high school graduate							
	High school graduate or higher	−0.251	0.266	0.892	0.345	0.778	0.462	1.310
Income	Low							
	High	0.101	0.192	0.275	0.600	1.106	0.759	1.613
Region	City(-si)							
	Province(-do)	0.044	0.184	0.057	0.812	1.045	0.728	1.499
Diabetes mellitus	Normal							
	Impaired fasting glucose	0.486	0.236	4.247	0.039	1.626	1.024	2.582
	Diabetes mellitus	0.404	0.333	1.467	0.226	1.497	0.779	2.877
Hypertension	Normal							
	High	−0.355	0.251	1.998	0.158	0.701	0.428	1.147
WBC count		0.020	0.058	0.113	0.737	1.020	0.910	1.143
Metabolic syndrome	No							
	Yes	−0.073	0.213	0.116	0.733	0.930	0.612	1.413
Toothbrushing yesterday	No							
	Yes	−0.435	1.044	0.174	0.677	0.647	0.084	5.006

BMI, Body mass index; WBC, white blood cell.

**Table 3:** Odds ratios, along with their corresponding 95% confidence intervals and *p*-values, for dental caries among male participants.

Variable	Level	Estimate	Standard Error	Wald Chi-Square	*p*-Value	OR	95% CI for OR	
Milk Amount	Low							
	High	0.478	0.2485	3.699	0.054	1.613	0.991	2.625
Age		0.021	0.012	2.966	0.085	1.022	0.997	1.047
Smoking	Current							
	Ex-	−0.240	0.260	0.849	0.357	0.787	0.472	1.310
	Non-	0.380	0.330	1.326	0.250	1.462	0.766	2.789
Drinking	Non-							
	Light-medium	0.123	0.279	0.195	0.659	1.131	0.655	1.953
	Heavy	0.112	0.301	0.138	0.710	1.118	0.620	2.017
BMI		−0.006	0.041	0.022	0.882	0.994	0.918	1.076
Regular physical exercise (average number of days per week)		−0.030	0.082	0.136	0.712	0.970	0.827	1.139
Education	Lower than high school graduate							
	High school graduate or higher	0.090	0.344	0.068	0.794	1.094	0.557	2.149
Income	Low							
	High	−0.063	0.228	0.076	0.783	0.939	0.601	1.468
Region	City(-si)							
	Province(-do)	−0.095	0.226	0.176	0.675	0.910	0.584	1.416
Diabetes	Normal							
	Impaired fasting glucose	0.269	0.271	0.981	0.322	1.308	0.769	2.227
	Diabetes mellitus	0.193	0.402	0.231	0.631	1.213	0.552	2.665
Hypertension	Normal							
	High	−0.557	0.319	3.046	0.081	0.573	0.306	1.071
WBC count		0.005	0.078	0.005	0.944	1.005	0.864	1.170
Metabolic syndrome	No							
	Yes	0.079	0.283	0.078	0.780	1.082	0.622	1.883
Toothbrushing yesterday	No							
	Yes	−12.178	0.563	468.305	<.0001	<0.001	<0.001	<0.001

BMI, Body mass index; WBC, white blood cell.

**Table 4:** The odds ratios for dental caries in female participants, along with their associated 95% confidence intervals and *p*-values.

Variable	Level	Estimate	Standard Error	Wald Chi-Square	*p*-Value	OR	95% CI for OR	
Milk Amount	Low							
	High	0.623	0.2436	6.542	0.011	1.865	1.157	3.006
Age		−0.038	0.019	4.108	0.043	0.963	0.928	0.999
Smoking	Current							
	Ex-	0.456	0.825	0.305	0.581	1.577	0.313	7.950
	Non-	0.376	0.655	0.329	0.566	1.456	0.404	5.251
Drinking	Non-							
	Light-medium	−0.760	0.309	6.057	0.014	0.468	0.255	0.857
	Heavy	0.900	0.636	2.001	0.157	2.460	0.707	8.562
BMI		0.022	0.050	0.194	0.660	1.022	0.927	1.127
Regular physical exercise (average number of days per week)		0.148	0.101	2.124	0.145	1.003	0.885	1.136
Education	Lower than high school graduate							
	High school graduate or higher	−0.954	0.355	7.213	0.007	1.159	0.950	1.413
Income	Low							
	High	0.605	0.343	3.109	0.078	1.831	0.935	3.588
Region	City(-si)							
	Province(-do)	0.313	0.277	1.274	0.259	1.367	0.794	2.352
Diabetes mellitus	Normal							
	Impaired fasting glucose	1.113	0.398	7.824	0.005	3.042	1.395	6.634
	Diabetes mellitus	0.784	0.596	1.730	0.189	2.191	0.681	7.051
Hypertension	Normal							
	High	0.201	0.413	0.237	0.626	1.223	0.544	2.747
WBC		0.049	0.082	0.357	0.550	1.050	0.895	1.232
Metabolic syndrome	No							
	Yes	−0.253	0.344	0.543	0.461	0.776	0.396	1.523
Toothbrushing yesterday	No							
	Yes	1.009	1.056	0.913	0.339	2.742	0.346	21.718

**Table 5:** Subgroup analysis of dental caries in both males and females, stratified by their levels of milk consumption.

Gender	Dental Caries	Amount of Milk Consumption		*p*-Value
		Low	High	
Male	No	53 (15.1)	116 (12.7)	0.2719
	Yes	332 (84.9)	834 (87.3)	
Female	No	50 (7.79)	75 (6.0)	0.1906
	Yes	572 (92.39)	1300 (94.0)	

## Data Availability

This publication encompasses all the data generated or analyzed during the course of this study.

## Supplementary Materials

The following supporting information can be downloaded at https://www.mdpi.com/article/10.3390/medicina60060967/s1. Table S1: Multiple linear regression results when response variable is the number of teeth experienced dental caries; Table S2: Logistic regression results with additional interaction effects with variables found to be significant in the main effect model (Model2-whole); Table S3: Logistic regression results with all possible interaction effects (Model3-whole); Table S4: Logistic regression results with additional interaction effects with variables found to be significant in the main effect model for the male population (Model2-male); Table S5: Logistic regression results with all possible interaction effects for the male population (Model3-male); Table S6: Logistic regression results with additional interaction effects with variables found to be significant in the main effect model for the female population (Model2-female); Table S7: Logistic regression results with all possible interaction effects for the female population (Model3-female); Table S8: Likelihood ratio test results.
