# Supplementary material for: Milk Consumption and Its Association with Dental Caries: Gender-Specific Insights from the Korea National Health and Nutrition Examination Survey (2013–2015)
Source: Medicina (Kaunas). 2024 Jun 11;60(6):967. doi: 10.3390/medicina60060967 (PMC11205343; doi:10.3390/medicina60060967)
Supplement: Supplementary file 1 [file medicina-60-00967-s001.zip › medicina-2977776-supplementary.pdf]

Supplementary Table S1. Multiple linear regression results when response variable is the number of teeth experienced dental caries.

Variable	Level	Estimate	Standard Error	t value	*p*-Value	95% CI for OR	
Milk Amount	low	.	.	.	.	.	.
	high	0.3517	0.2814	1.2500	0.2130	-0.204	0.907
Age		-0.0022	0.0132	-0.1700	0.8685	-0.028	0.024
Gender	Female	.	.	.	.	.	.
	Male	-1.5871	0.2989	-5.3100	<.0001	-2.177	-0.997
Smoking	currently	.	.	.	.	.	.
	past	-0.4562	0.3911	-1.1700	0.2451	-1.229	0.316
	none	-0.4649	0.3247	-1.4300	0.1542	-1.106	0.176
Drinking	none	.	.	.	.	.	.
	light-medium	-0.4426	0.3052	-1.4500	0.1490	-1.045	0.160
	heavy	-0.3930	0.3348	-1.1700	0.2422	-1.054	0.268
BMI		-0.0807	0.0365	-2.2100	0.0283	-0.153	-0.009
Regular physical exercise (average number of days per week)		0.0386	0.1013	0.3800	0.7038	-0.161	0.239
Education	Lower than high school graduate	.	.	.	.	.	.
	High school graduate or higher	-0.5882	0.3509	-1.6800	0.0956	-1.281	0.105
Income	low	.	.	.	.	.	.
	high	0.2799	0.2664	1.0500	0.2949	-0.246	0.806
Region	City(-si)	.	.	.	.	.	.
	Province(-do)	-0.2795	0.2715	-1.0300	0.3048	-0.816	0.257
Diabetes mellitus	Normal	.	.	.	.	.	.
	Impaired fasting glucose	0.5619	0.2803	2.0000	0.0466	0.008	1.115
	Diabetes mellitus	0.5313	0.4368	1.2200	0.2256	-0.331	1.394
Hypertension	normal	.	.	.	.	.	.
	high	-0.3005	0.3520	-0.8500	0.3945	-0.996	0.395

WBC		-0.0188	0.0922	-0.2000	0.8386	-0.201	0.163
Metabolic Syndrom	no	.	.	.	.	.	.
	yes	-0.0060	0.2825	-0.0200	0.9832	-0.564	0.552
Number of brushing yesterday		-0.1772	0.1296	-1.3700	0.1734	-0.433	0.079

Supplementary Table S2. Logistic regression results with additional interaction effects with variables found to be significant in the main effect model (Model2-whole).

Variable	Level	Estimate	Standard Error	Wald Chi-Square	*p*-Value	OR	95% CI for OR	
Milk Amount	Low	.	.	.	.	.	.	.
	High	0.574	0.258	4.964	0.026	1.775	1.072	2.941
Age		0.003	0.010	0.092	0.761	1.003	0.984	1.022
Gender	Female	.	.	.	.	.	.	.
	Male	-0.552	0.316	3.044	0.081	0.576	0.310	1.071
Smoking	Current	.	.	.	.	.	.	.
	Ex-	-0.175	0.237	0.541	0.462	0.840	0.527	1.337
	Non-	0.223	0.274	0.668	0.414	1.250	0.731	2.137
Drinking	Non-	.	.	.	.	.	.	.
	Light-medium	-0.190	0.215	0.785	0.376	0.827	0.543	1.259
	Heavy	0.014	0.260	0.003	0.956	1.015	0.609	1.689
BMI		0.005	0.032	0.025	0.875	1.005	0.945	1.069
Regular physical exercise (average number of days per week)		0.005	0.064	0.006	0.937	1.005	0.887	1.139
Education	Lower than high school graduate	.	.	.	.	.	.	.
	High school graduate or higher	-0.265	0.270	0.964	0.326	0.768	0.453	1.302
Income	Low	.	.	.	.	.	.	.
	High	0.101	0.191	0.278	0.598	1.106	0.760	1.610
Region	City(-si)	.	.	.	.	.	.	.
	Province(-do)	0.042	0.183	0.052	0.820	1.042	0.728	1.493
Diabetes mellitus	Normal	.	.	.	.	.	.	.

	Impaired fasting glucose	0.283	0.393	0.517	0.472	1.327	0.614	2.866
	Diabetes mellitus	0.333	0.480	0.480	0.489	1.395	0.544	3.574
Hypertension	Normal	.	.	.	.	.	.	.
	High	-0.358	0.249	2.078	0.149	0.699	0.429	1.138
WBC		0.021	0.058	0.132	0.716	1.021	0.911	1.145
Metabolic syndrome	No	.	.	.	.	.	.	.
	Yes	-0.062	0.214	0.084	0.772	0.940	0.617	1.430
Toothbrushing yesterday	No	.	.	.	.	.	.	.
	Yes	-0.433	1.047	0.171	0.679	0.648	0.083	5.050
Milk amount*Sex		.	.	.	.	.	.	.
	high *Male	-0.250	0.353	0.501	0.479	0.779	0.390	1.557
Milk amount* Diabetes mellitus	high *Normal	.	.	.	.	.	.	.
	high *Impaired fasting glucose	0.344	0.477	0.520	0.471	1.410	0.554	3.591
	high *Diabetes mellitus	0.109	0.613	0.031	0.860	1.115	0.335	3.709

Supplementary Table S3. Logistic regression results with all possible interaction effects (Model3-whole).

Variable	Level	Estimate	Standard Error	Wald Chi-Square	*p*-Value	OR	95% CI for OR	
Milk Amount	low	.	.	.	.	.	.	.
	high	12.623	2.327	29.436	<.0001	303519.155	3175.003	29015362.817
Age		0.012	0.018	0.453	0.501	1.012	0.978	1.047
Gender	Female	.	.	.	.	.	.	.
	Male	-0.631	0.351	3.225	0.073	0.532	0.267	1.059
Smoking	currently	.	.	.	.	.	.	.
	past	0.245	0.396	0.385	0.535	1.278	0.589	2.775
	none	0.332	0.385	0.743	0.389	1.393	0.655	2.961
Drinking	none	.	.	.	.	.	.	.
	light-medium	-0.322	0.355	0.825	0.364	0.724	0.361	1.453
	heavy	-0.025	0.383	0.004	0.948	0.975	0.460	2.066
BMI		0.033	0.040	0.670	0.413	1.033	0.956	1.117

Regular physical exercise (average number of days per week)		-0.060	0.122	0.246	0.620	0.941	0.741	1.195
Education	Lower than high school graduate	.	.	.	.	.	.	.
	High school graduate or higher	-0.208	0.393	0.280	0.596	0.812	0.376	1.754
Income	low	.	.	.	.	.	.	.
	high	0.218	0.345	0.399	0.528	1.243	0.632	2.445
Region	City(-si)	.	.	.	.	.	.	.
	Province(-do)	0.269	0.268	1.012	0.315	1.309	0.775	2.212
Diabetes mellitus	Normal	.	.	.	.	.	.	.
	Impaired fasting glucose	0.306	0.399	0.588	0.443	1.357	0.622	2.964
	Diabetes mellitus	0.341	0.499	0.468	0.494	1.406	0.529	3.736
Hypertension	Normal	.	.	.	.	.	.	.
	High	-0.596	0.338	3.112	0.078	0.551	0.284	1.068
WBC		0.049	0.097	0.255	0.613	1.050	0.869	1.269
Metabolic Syndrome	no	.	.	.	.	.	.	.
	yes	-0.262	0.367	0.512	0.474	0.769	0.375	1.578
Number of	no					1.000	1.000	1.000
brushing yesterday	yes	0.520	1.202	0.187	0.665	1.682	0.160	17.730
Milk Amount*age	high	-0.014	0.022	0.383	0.536	0.986	0.944	1.030
Milk Amount*gender	Female	.	.	.	.	.	.	.
	Male	-0.145	0.466	0.097	0.756	0.865	0.347	2.157
Milk Amount*Smoking	currently	.	.	.	.	.	.	.
	past	-0.661	0.492	1.805	0.179	0.516	0.197	1.354
	none	-0.186	0.501	0.137	0.711	0.831	0.311	2.217
Milk Amount*Drinking	none	.	.	.	.	.	.	.
	light-medium	0.163	0.469	0.120	0.729	1.177	0.469	2.951
	heavy	0.036	0.541	0.004	0.947	1.036	0.359	2.991
Milk Amount*BMI		-0.046	0.059	0.605	0.437	0.955	0.850	1.073

Milk Amount* Regular physical exercise (average number of days per week)		0.110	0.154	0.510	0.475	1.117	0.825	1.511
Milk Amount*Education	high*Lower than high school graduate	.	.	.	.	.	.	.
	high*High school graduate or higher	-0.108	0.520	0.043	0.835	0.898	0.324	2.485
Milk Amount*Income		.	.	.	.	.	.	.
	high*high	-0.167	0.421	0.157	0.692	0.846	0.371	1.931
Milk Amount*Region	high*City(-si)	.	.	.	.	.	.	.
	high*Province(-do)	-0.370	0.354	1.093	0.296	0.691	0.345	1.382
Milk Amount*Diabetes mellitus	high*Normal	.	.	.	.	.	.	.
	high*Impaired fasting glucose	0.316	0.491	0.414	0.520	1.372	0.524	3.591
	high*Diabetes mellitus	0.061	0.646	0.009	0.925	1.063	0.300	3.770
Milk Amount* Hypertension	high*Normal	.	.	.	.	.	.	.
	high*High	0.442	0.556	0.634	0.426	1.556	0.524	4.623
Milk Amount*WBC		-0.039	0.114	0.116	0.734	0.962	0.770	1.202
Milk Amount*Metabolic Syndrome	high *no	.	.	.	.	.	.	.
	high *yes	0.323	0.478	0.457	0.499	1.382	0.541	3.528
Milk Amount* Toothbrushing yesterday	high *no							
	high *yes	-10.127	1.345	56.672	<.0001	0.000	0.000	#############

Supplementary Table S4. Logistic regression results with additional interaction effects with variables found to be significant in the main effect model for the male population (Model2-male).

Variable	Level	Estimate	Standard Error	Wald Chi-Square	*p*-Value	OR	95% CI for OR	
Milk Amount	low	.	.	.	.	.	.	.
	high	0.655	1.999	0.108	0.743	1.926	0.038	96.829
Age		0.036	0.022	2.567	0.109	1.036	0.992	1.082
Smoking	currently	.	.	.	.	.	.	.
	past	-0.237	0.260	0.833	0.362	0.789	0.474	1.313
	none	0.368	0.332	1.228	0.268	1.445	0.754	2.770
Drinking	none	.	.	.	.	.	.	.
	light-medium	0.096	0.281	0.116	0.734	1.100	0.634	1.910
	heavy	0.098	0.304	0.104	0.747	1.103	0.608	2.002
BMI		-0.007	0.041	0.033	0.855	0.993	0.916	1.075
Regular physical exercise (average number of days per week)		-0.026	0.081	0.106	0.745	0.974	0.832	1.141
Education	Lower than high school graduate	.	.	.	.	.	.	.
	High school graduate or higher	0.094	0.344	0.074	0.786	1.098	0.560	2.155
Income	low	.	.	.	.	.	.	.
	high	-0.043	0.235	0.034	0.854	0.958	0.605	1.517
Region	City(-si)	.	.	.	.	.	.	.
	Province(-do)	-0.111	0.231	0.229	0.632	0.895	0.570	1.408
Diabetes mellitus	Normal	.	.	.	.	.	.	.
	Impaired fasting glucose	0.262	0.272	0.932	0.334	1.300	0.763	2.213
	Diabetes mellitus	0.156	0.405	0.149	0.700	1.169	0.529	2.585
Hypertension	Normal	.	.	.	.	.	.	.
	High	-0.875	0.446	3.854	0.050	0.417	0.174	0.998
WBC		0.002	0.079	0.001	0.976	1.002	0.858	1.171
Metabolic Syndrome	no	.	.	.	.	.	.	.
	yes	0.091	0.282	0.103	0.748	1.095	0.630	1.904
Tooth brushing yesterday	no	.	.	.	.	.	.	.
	yes	-8.665	0.885	95.909	<.0001	0.000	0.000	0.001
Milk Amount*Age		-0.021	0.026	0.656	0.418	0.979	0.930	1.031

Milk Amount* Hypertension	normal	.	.	.	.	.	.	.
	high	0.527	0.654	0.649	0.421	1.693	0.470	6.097
Milk Amount*Tooth brushing yesterday	no	.	.	.	.	.	.	.
	yes	0.645	1.227	0.277	0.599	1.906	0.172	21.099

Supplementary Table S5. Logistic regression results with all possible interaction effects for the male population (Model3-male).

Variable	Level	Estimate	Standard Error	Wald Chi-Square	*p*-Value	OR	95% CI for OR	
Milk Amount	low	.	.	.	.	.	.	.
	high	2.149	3.234	0.442	0.506	8.577	0.015	4852.979
Age		0.038	0.023	2.784	0.095	1.039	0.993	1.087
Smoking	currently	.	.	.	.	.	.	.
	past	0.258	0.439	0.347	0.556	1.295	0.548	3.058
	none	0.910	0.615	2.188	0.139	2.484	0.744	8.290
Drinking	none	.	.	.	.	.	.	.
	light-medium	0.059	0.526	0.013	0.910	1.061	0.378	2.976
	heavy	0.206	0.469	0.192	0.661	1.229	0.490	3.083
BMI		0.011	0.062	0.032	0.857	1.011	0.896	1.141
Regular physical exercise (average number of days per week)		-0.070	0.159	0.192	0.661	0.932	0.682	1.274
Education	Lower than high school graduate	.	.	.	.	.	.	.
	High school graduate or higher	0.345	0.520	0.439	0.508	1.411	0.509	3.912
Income	low	.	.	.	.	.	.	.
	high	-0.072	0.466	0.024	0.877	0.930	0.373	2.320
Region	City(-si)	.	.	.	.	.	.	.
	Province(-do)	0.017	0.404	0.002	0.966	1.017	0.461	2.247
Diabetes mellitus	Normal	.	.	.	.	.	.	.
	Impaired fasting glucose	0.236	0.506	0.217	0.641	1.266	0.469	3.416

	Diabetes mellitus	0.175	0.633	0.077	0.782	1.191	0.345	4.120
Hypertension	Normal	.	.	.	.	.	.	.
	High	-0.881	0.466	3.575	0.059	0.414	0.166	1.033
WBC		0.039	0.135	0.083	0.773	1.040	0.798	1.355
Metabolic Syndrome	no	.	.	.	.	.	.	.
	yes	-0.169	0.473	0.127	0.722	0.845	0.334	2.135
Tooth brushing yesterday	no	.	.	.	.	.	.	.
	yes	-8.867	0.962	84.945	<.0001	0.000	0.000	0.001
Milk Amount*Age		-0.025	0.028	0.835	0.361	0.975	0.923	1.029
Milk Amount*Smoking	currently	.	.	.	.	.	.	.
	past	-0.776	0.535	2.108	0.147	0.460	0.161	1.312
	none	-0.790	0.697	1.285	0.257	0.454	0.116	1.778
Milk Amount*Drinking	none	.	.	.	.	.	.	.
	light-medium	0.032	0.680	0.002	0.963	1.032	0.272	3.915
	heavy	-0.173	0.664	0.068	0.795	0.841	0.229	3.089
Milk Amount*BMI		-0.028	0.083	0.109	0.741	0.973	0.826	1.146
Milk Amount* Regular physical exercise (average number of days per week)		0.070	0.198	0.125	0.723	1.073	0.728	1.582
Milk Amount*Education	Lower than high school graduate	.	.	.	.	.	.	.
	High school graduate or higher	-0.410	0.672	0.372	0.542	0.664	0.178	2.479
Milk Amount*Income	low	.	.	.	.	.	.	.
	high	0.024	0.540	0.002	0.965	1.024	0.356	2.947
Milk Amount*Region	City(-si)	.	.	.	.	.	.	.
	Province(-do)	-0.194	0.507	0.146	0.703	0.824	0.305	2.225
Milk Amount*Diabetes	Normal	.	.	.	.	.	.	.
	Impaired fasting glucose	0.081	0.604	0.018	0.893	1.085	0.332	3.543
	Diabetes mellitus	-0.017	0.814	0.001	0.983	0.983	0.199	4.849

Milk Amount* Hypertension	normal	.	.	.	.	.	.	.
	high	0.570	0.687	0.690	0.406	1.769	0.460	6.797
Milk Amount*WBC		-0.053	0.147	0.131	0.717	0.948	0.711	1.264
Milk Amount*Metabolic Syndrome	no	.	.	.	.	.	.	.
	yes	0.372	0.590	0.398	0.528	1.451	0.457	4.606
Milk Amount* Tooth brushing yesterday	no					1.000	1.000	1.000
	yes	0.907	1.285	0.498	0.480	2.476	0.200	30.711

Supplementary Table S6. Logistic regression results with additional interaction effects with variables found to be significant in the main effect model for the female population (Model2-female).

Variable	Level	Estimate	Standard Error	Wald Chi-Square	*p*-Value	OR	95% CI for OR	
Milk Amount	low	.	.	.	.	.	.	.
	high	-0.608	2.010	0.092	0.762	0.544	0.011	27.997
Age		-0.049	0.029	2.864	0.091	0.953	0.900	1.008
Smoking	currently	.	.	.	.	.	.	.
	past	0.359	0.820	0.191	0.662	1.432	0.287	7.142
	none	0.374	0.652	0.329	0.566	1.453	0.405	5.212
Drinking	none	.	.	.	.	.	.	.
	light-medium	-0.913	0.391	5.445	0.020	0.401	0.186	0.864
	heavy	0.258	0.750	0.119	0.731	1.294	0.298	5.624
BMI		0.022	0.049	0.203	0.653	1.022	0.928	1.126
Regular physical exercise (average number of days per week)		0.158	0.103	2.349	0.125	1.171	0.957	1.434
Education	Lower than high school graduate	.	.	.	.	.	.	.
	High school graduate or higher	-1.099	0.467	5.532	0.019	0.333	0.133	0.833
Income	low	.	.	.	.	.	.	.
	high	0.628	0.341	3.394	0.065	1.873	0.961	3.652
Region	City(-si)	.	.	.	.	.	.	.
	Province(-do)	0.317	0.274	1.332	0.249	1.373	0.802	2.350
Diabetes mellitus	Normal	.	.	.	.	.	.	.

	Impaired fasting glucose	0.420	0.463	0.821	0.365	1.522	0.614	3.774
	Diabetes mellitus	0.515	0.679	0.576	0.448	1.674	0.442	6.338
Hypertension	Normal	.	.	.	.	.	.	.
	High	0.198	0.426	0.215	0.643	1.218	0.529	2.806
WBC		0.051	0.082	0.377	0.540	1.052	0.895	1.236
Metabolic Syndrome	no	.	.	.	.	.	.	.
	yes	-0.253	0.345	0.538	0.463	0.777	0.395	1.527
Tooth brushing yesterday	no	.	.	.	.	.	.	.
	yes	1.291	1.126	1.314	0.252	3.635	0.400	33.028
Milk Amount*Age		0.013	0.034	0.148	0.700	1.013	0.948	1.084
Milk Amount*Drinking	none	.	.	.	.	.	.	.
	light-medium	0.274	0.549	0.250	0.617	1.316	0.449	3.859
	heavy	1.250	1.343	0.867	0.352	3.491	0.251	48.521
Milk Amount*Education	Lower than high school graduate	.	.	.	.	.	.	.
	High school graduate or higher	0.210	0.662	0.101	0.751	1.234	0.337	4.514
Milk Amount*Diabetes mellitus	Normal	.	.	.	.	.	.	.
	Impaired fasting glucose	1.899	0.831	5.222	0.022	6.676	1.310	34.017
	Diabetes mellitus	0.612	0.885	0.477	0.490	1.843	0.325	10.451

Supplementary Table S7. Logistic regression results with all possible interaction effects for the female population (Model3-female).

Variable	Level	Estimate	Standard Error	Wald Chi-Square	*p*-Value	OR	95% CI for OR	
Milk Amount	low	.	.	.	.	.	.	.
	high	9.753	3.203	9.274	0.002	17207.489	32.324	9160323.018
Age		-0.050	0.028	3.228	0.072	0.951	0.900	1.005
Smoking	currently	.	.	.	.	.	.	.
	past	0.438	1.474	0.088	0.767	1.549	0.086	27.864
	none	-0.776	0.901	0.742	0.389	0.460	0.079	2.690

Drinking	none	.	.	.	.	.	.	.
	light-medium	-1.170	0.411	8.103	0.004	0.310	0.139	0.695
	heavy	-0.225	0.691	0.106	0.745	0.798	0.206	3.094
BMI		0.054	0.053	1.046	0.307	1.056	0.951	1.172
Regular physical exercise (average number of days per week)		0.014	0.167	0.007	0.933	1.014	0.731	1.408
Education	Lower than high school graduate	.	.	.	.	.	.	.
	High school graduate or higher	-1.275	0.506	6.346	0.012	0.279	0.104	0.753
Income	low	.	.	.	.	.	.	.
	high	1.364	0.578	5.565	0.018	3.910	1.259	12.142
Region	City(-si)	.	.	.	.	.	.	.
	Province(-do)	0.606	0.389	2.429	0.119	1.833	0.855	3.929
Diabetes mellitus	Normal	.	.	.	.	.	.	.
	Impaired fasting glucose	0.469	0.469	1.004	0.317	1.599	0.638	4.005
	Diabetes mellitus	0.605	0.713	0.719	0.397	1.831	0.452	7.407
Hypertension	Normal	.	.	.	.	.	.	.
	High	-0.024	0.560	0.002	0.966	0.976	0.326	2.923
WBC		-0.019	0.137	0.019	0.891	0.981	0.751	1.283
Metabolic Syndrom	no	.	.	.	.	.	.	.
	yes	-0.079	0.479	0.027	0.869	0.924	0.361	2.364
Numbe of brushing yesterday	no	.	.	.	.	.	.	.
	yes	2.614	1.162	5.059	0.025	13.651	1.399	133.155
Milk Amount*Age		0.015	0.037	0.153	0.696	1.015	0.944	1.091
Milk Amount*Smoking	currently	.	.	.	.	.	.	.
	past	-0.132	1.737	0.006	0.940	0.877	0.029	26.368
	none	1.750	1.133	2.386	0.122	5.754	0.625	53.005
Milk Amount*Drinking	none	.	.	.	.	.	.	.
	light-medium	0.534	0.564	0.898	0.343	1.706	0.565	5.147
	heavy	1.952	1.388	1.980	0.159	7.045	0.464	106.890

Milk Amount*BMI		-0.070	0.092	0.589	0.443	0.932	0.779	1.116
Milk Amount* Regular physical exercise (average number of days per week)		0.224	0.227	0.970	0.325	1.251	0.801	1.951
Milk Amount*Education	Lower than high school graduate	.	.	.	.	.	.	.
	High school graduate or higher	0.426	0.740	0.332	0.565	1.531	0.359	6.527
Milk Amount*Income	low	.	.	.	.	.	.	.
	high	-1.062	0.703	2.284	0.131	0.346	0.087	1.371
Milk Amount*Region	City(-si)	.	.	.	.	.	.	.
	Province(-do)	-0.530	0.496	1.143	0.285	0.589	0.223	1.555
Milk Amount*Diabetes	Normal	.	.	.	.	.	.	.
	Impaired fasting glucose	1.877	0.800	5.507	0.019	6.533	1.363	31.319
	Diabetes mellitus	0.555	0.899	0.382	0.537	1.742	0.299	10.151
Milk Amount* Hypertension	normal	.	.	.	.	.	.	.
	high	0.454	0.774	0.344	0.558	1.574	0.346	7.171
Milk Amount*WBC		0.111	0.174	0.407	0.523	1.118	0.794	1.572
Milk Amount*Metabolic Syndrome	no	.	.	.	.	.	.	.
	yes	-0.288	0.642	0.202	0.654	0.750	0.213	2.640
Milk Amount* Tooth brushing yesterday	no	.	.	.	.	.	.	.
	yes	-11.350	1.236	84.367	<.0001	0.000	0.000	0.000

Supplementary Table S8. Likelihood ratio test results. *p*-Values for the comparison of each combination are shown. Large *p*-value indicates that restrictive (conservative) model is enough to explain the data.

p-Value	M2	M3

M1	Whole population	1	1
	Male population	1	1
	Female Population	1	1
